# Factors Associated with Anger among Male Adolescents in Western Iran: An Application of Social Cognitive Theory

**DOI:** 10.5539/gjhs.v7n6p338

**Published:** 2015-05-20

**Authors:** Mohammad Sadegh Abedzadeh Zavareh, Shamsaddin Niknami, Ali Reza Hidarnia

**Affiliations:** 1 Health Education Department, Faculty of Medical Sciences, Tarbiat Modares University, Tehran, Iran

**Keywords:** anger, social cognitive theory, adolescent, student, Iran

## Abstract

**Introduction:**

Anger can be defined a natural emotional response that is gradually formed to protect us in dealing with threats, damages, assaults, and failures; while hatred is a change of attitude which is built following the persistence of anger towards a subject or an individual. One of the main reasons of adolescents’ reference to the counseling centers is their anger accompanied by violence.

**Objective:**

This study aims to determine the social cognitive factors associated with anger among a population of adolescents in the west of Iran based on the social cognitive theory.

**Methodology:**

Samples were selected based on multi-stage cluster sampling. Method including the first and the second-grade male high school students from Ilam town (N=360). The Spielberger’s anger questionnaire (STAXI 2) and a self-designed questionnaire based on Bandura’s social cognitive theory were employed as the data collection instruments in the present study.

**Results:**

Of the selected population, 200 students were the first-grade and 160 students were the second-grade students. 135 students were the first child of the family, 150 students were the second or the third birth, and 75 students were the fifth or above in their families. Descriptive tests and correlation analysis were used to conduct the statistical analysis. Although there was a significant and inverse relationship between all the components of the theory and anger, the strongest relationship was seen in self-efficacy (-0.585) and the weakest relationship was seen in modeling (-0.297).

**Discussion and Conclusion:**

If was concluded that helping people to know their abilities and have a better personal judgment in this case, can influence their anger control. In addition, the process of stress management can effectively increase an individual’s emotional coping.

## 1. Introduction

Anger can be defined as a natural emotional response that is gradually formed to protect us in dealing with threats, damages, assaults, and failures; while hatred is a change of attitude which is built following the persistence of anger towards a subject or an individual. Additionally, aggression is a visible behavior that is usually aimed at harming others or something (Nicolet, 2004). Anger can also be considered as a satisfying emotion or a devastating one. It activates our internal systems and provides us to face with the potential risks surrounding (Taylor & Novaco, 2005).

Anger related issues such as coping behavior, hostility and aggression are among the main reasons behind the reference of adolescents, the young adults and even children to mental health counseling centers (Maleki, Fallahi, Rahgooi & Rahgozar, 2011).

Several researchers have claimed that an individual’s inability to express their feelings effectively has a significant relationship with anger and this issue can be taken into account as one of the leading factors for addiction reappearance (Schonfeld et al., 2000). Similarly, some findings have revealed that there is a significant relationship between the amount of external anger demonstration with the abuse of alcohol, drugs and tobacco. Anger has also been specified as a predictor factor for drug abuse such as cocaine (Kirby, Lamb, Iguchi, Husband & Platt, 1995; Wills, Sandy, Yaeger, Cleary & Shinar, 2001). As well, the environmental stresses directly affect delinquency and aggression, and anger caused by stressful environments is in the form of a mediator for risky behaviors (Bakhshipour R., Mahmood Alilou, & Irani, 2008; Clark, Wood, Cornelius, Bukstein & Martin, 2003).

Anger has also been recognized as one of the factors affecting students’ aggression (Maleki et al., 2011) as well as an effective factor which has an impact on the interaction between parents and children (Sanders et al., 2004).

In many studies, researchers have mentioned the specification of the effective role of surrounding factors on adolescent anger such as socio-economic, political, and cultural problems alongside strengthening and empowering adolescents to cope with anger and have internal management among the strategies which can have an effect on anger control and its development and ultimately reduce aggressive and antisocial behavior (Hashemian, Shafieabadi, & Soudani, 2008).

As a result, it is concluded that the effects of weakness and inability in anger management is higher than personal inconveniences and damages to the interpersonal relationships. These effects can also lead to disturbance in public health, appearance of public incompatibility, and the harmful consequences of aggressive behaviors. In case this powerful emotion is not controlled properly, it can prevent overall success and optimal functioning of individuals, groups and communities (Naveedy, 2009).

Numerous studies have been conducted in conjunction with anger, and a number of factors such as self-awareness, social support, behavioral factors, self-control, self-efficacy, relaxation, self-monitoring and as a whole cognitive-behavioral factors have been cited as the factors which have an impact on it (Lombardo, Tan, Jensen & Anderson, 2005; Mohammadiarya et al., 2012; Shokoohi-Yekta, Zamani, & Parand, 2010; Shokoohi-Yekta, Zamani, Parand, Ayazi & Lotfi, 2011).

In the present study, social cognitive theory was employed to consider the above-mentioned factors. This theory, designed and established by Albert Bandura, assumes that human behavior can be illustrated by a two-directional triple causation. These factors include behavioral factors, personal factors, and environmental ones (Glanz, Rimer, & Viswanath, 2008). Since these factors are considered and employed in this theory, the main objective of the present study is to determine the social cognitive factors associated with anger among adolescents in western Iran based on the social cognitive theory.

## 2. Materials and Method

### 2.1 Subject

All the first and the second-grade male high school students comprised the statistical population of the present study. The participants (N= 360) were from 4 high schools selected through cluster sampling among 16 public male high schools. Samples were selected using random and classified sampling (based on the educational years) between the first and the second-grade students. The inclusion criteria were studying at the first and the second year of study in public high schools, having no mental illness certified by a physician, taking no psychiatric medications, and having consent to participate in the project (all the participants in the project endorsed a written consent). Those who did not have the mentioned conditions were excluded. In terms of the ethical approval, this project was confirmed by the ethics committee of Tarbiat Modares University, Tehran.

### 2.2 Instruments

Two questionnaires including the Persian version of Spielberger anger test (STAXI 2) and a self-designed questionnaire based on the social cognitive theory were administered. The anger test (STAXI 2) with 57 items was prepared by Spielberger in 1999. This questionnaire is composed of 6 scales (state anger, trait anger, anger expression out, anger expression in, anger control out, and anger control in); and 5 sub-scales (feeling angry, fill like expression anger verbally, fill like expression anger physically, angry temperament, and angry reaction) and general anger expression index. Responses to the Spielberger’s anger questionnaire were based on a four-scale degree ranging from (never=1) to (a lot=4), and also, (rarely=1) to (always=4).Asghari Moghadam et al. (2011), determined the validity and reliability of Persian version of Spielberger’s anger questionnaire. Internal consistency for state anger, trait anger and general anger index in their study were 0.91, 0.82, and 0.81 respectively. In order to measure the constructs of the social cognitive theory in the self-designed questionnaire, the number of questions and the Cronbach’s alpha coefficients are summarized in Table 1.

**Table 1:** Internal consistency of social cognitive variables

Variables	Number of Items	Cronbach’s alpha (α)	Define variables
Knowledge	4	0.573	Learning facts and gaining insights
Modeling	5	0.618	observational learning
Outcome expectancies	5	0.768	Value a person places on the probable outcomes that result from performing a behavior
Outcome expectation	5	0.723	Anticipation of the probable outcomes that would ensue as a result of engaging in the behavior under discussion
Social support	4	0.85	Social circumstances or conditions that surround a person
emotional coping	4	0.76	Techniques employed by the person to control the emotional and physiological states associated with acquisition of a new behavior
self-efficacy	4	0.72	Confidence in one’s ability to pursue a behavior
goal setting or self-control	4	0.787	Setting goals and developing plans to accomplish chosen behavior

The questionnaire of social cognitive theory contained several construct amongst them, the knowledge construct was in the form of true, false and I don’t know; and the others were in the form of five-choice Likert-scale ranging from completely disagree to completely agree. The demographic questions included grade, field of study, family size, birth order, age, father’s education, mother’s education, and father’s and mother’s occupations.

The questionnaires were completed from November 13th to November 30th, 2014. For data collection the researcher referred to 4 high school of Ilam town and after coordinating to school principal the questionnaire were distributed between first and second year high school students. Before completion the questionnaire by the students about ten minutes the researcher explained about the aims of the study and how to complete the questionnaires.

Descriptive statistics and inter-correlation analysis tests were used, to analyze the data using SPSS version 16.

## 3. Results

All the participants in the first and the second-grade high school students aged between 15 and 16 years. 10% of students’ fathers and 16.1% of their mothers were illiterate. About 33.1% of families were relatively populated (6 and above). It was also revealed that 37.5% of the participants were the first child of the family. About 70.3% of students were 15 years old, and the rest (29.7%) were 16 years old (Table 2).

**Table 2:** Socio demographic characteristics of study subjects

*Characteristics*	*N*	*%*
Age		
15	253	70.3
16	107	29.7
Students Educational Grades		
1th	200	55.6
2th	160	44.4
Father educational level		
Illiterate	36	10
Under diploma	188	52.2
Diploma and higher	136	37.8
Mother educational level		
Illiterate	58	16.1
Under diploma	242	67.2
Diploma and higher	60	16.7
Family dimension		
3	9	2.5
4-5	232	64.4
6 and highest	119	33.1
Rank of birth		
1th	135	37.5
2&3th	150	41.7
4^th^ and highest	75	20.8

All of the 8 measured scales had a significant and inverse relationship with anger so that the strongest and the weakest relationships were between anger and self-efficacy and anger and modeling, respectively (Table 3). Also there was a significant relationship between the 8 components (the strongest relationship was between self-efficacy and emotional coping and the weakest relationship was between knowledge and social support).

**Table 3:** Means, standard deviations, and inter-correlations for anger and social cognitive variables

Variables	1	2	3	4	5	6	7	8	9	Mean	SD
1. Anger (AX.Index)	-									40.12	1.37
2. Modeling	-.279**	-								12.7	3.51
3. Social support (Environment)	-.358**	.498**	-							9.83	3.32
4.Outcome expectation	-.410**	.341**	.447**	-						15.83	3.35
5.Self – control	-.497**	.244**	.334**	.492**	-					11.03	2.93
6.Emotional coping	-.547**	.307**	.404**	.365**	.453**	-				8.93	3.64
7.Outcome expectancy	-.435**	.354**	.426**	.581**	.543**	.434**	-			13.44	3.99
8.Knowledge	-.423**	.179**	.258**	.349**	.391**	.231**	.374**	-		5.19	1.91
9.Self – efficacy	-.585**	.414**	.431**	.431**	.493**	.499**	.456**	.323**	-	8.69	2.76

** Correlation is significant at the 0.01 level (2-tailed).

## 4. Discussion and Conclusion

According to the findings of the current study, there was a significant and inverse relationship between the general expression of anger and all the components of the social cognitive theory.

There is a significant relationship between the anger scale and modeling. Although this relationship is weaker in comparison with other components, it was revealed that an individual directly copies behaviors, and others’ behaviors have instigating effects on a person. Some researcher have stressed on such issue and revealed that the modeling can have an impact on students’ anger (Gansle, 2005).

Likewise, there was a significant relationship between social support and anger. In this way, having social support can be highly helpful to the creation of a new behavior, or quitting a wrong behavior (anger). In some other studies the family environment was mentioned that can influence the trait anger (Lopez & Thurman, 1993). Moreover, in one study on HIV positive patients it was confirmed that social support reduces the amount of anger (Whitehead, Hearn, & Burrell, 2014), while in another study carried out on patients under angiographic treatment it was revealed that there was a significant and inverse relationship between the social support and anger expression (León, Nouwen, Sheffield, Jaumdally & Lip, 2010). It has been demonstrated that people who were supported by religious communities have gone through lesser anger (Márquez-González, López, Romero-Moreno, & Losada, 2012). Furthermore some other researchers showed a significant relationship between social support and more social ties with anger (Ha & Ingersoll-Dayton, 2011).

It was revealed that there was a significant relationship between outcome expectation and the anger. The outcome expectation here refers to the physical consequences (positive and negative consequences), social approval or disapproval and their positive or negative evaluation (Bandura, 2004). Findings provided by Mauss, highlighted that anger control was in association with the negative results. There was similarly a significant relationship between emotion control and the positive results; and as it was noted, there was a relationship between the emotion control and anger control. In fact, the positive results influence anger intermediately (Mauss, Cook, & Gross, 2007).

As it was shown in Table 3, there was a significant relationship between the anger and self-control, self-control implies that a person sets several primary and final goals and tries to reduce anger. Studies by Gilliom and others are consistent with the findings of the present study (Davey, Day, & Howells, 2005).

Additionally, Novako in a series of studies showed that self-control can have some bearing on the anger of 17 to 42 year-old individuals (Novaco, 1976). Beck also concluded that self-control has an impact on anger (Beck & Fernandez, 1998).

According to the findings illustrated in Table 3, there was a significant relationship between the anger and emotional coping. The emotional coping refers to strategies employed by an individual in order to control emotional and physiological states in association with showing a behavior. It was revealed that the emotional state can be influential to self-control. This means that the emotional state can indirectly influence the anger (Singh et al., 2011). Another studies showed that there is a significant relationship between emotion control and anger (Garnefski et al., 2002; Martin & Dahlen, 2005). It was also shown that there is a relationship between relaxation and aggression (Novaco, 1976).

There was a significant relationship between outcome expectancies and anger. The consequence value here means the value an individual places on the possible consequences of performing a particular behavior. Some researchers carried out a study on mouth and teeth hygiene of pregnant women and showed that the more the benefits and the value of a behavior for an individual were proportional to the higher probability of showing that behavior. These findings were consistent with the results of the present study (Shamsi et al., 2014).

There is a significant relationship between anger and knowledge. This may possibly originate from this point that the more an individual knows about anger and is aware of it, the more their ability for and motivation towards anger control and the importance of control. Mohammadiarya in a study showed that self-knowledge can be effective on aggression and anger management which was in consonance with the results of the current study (Mohammadiarya, et al., 2012).

According to the findings shown in Table 3, there was a significant relationship between the anger and the self-efficacy. The self-efficacy here means an individual’s judgment about their abilities in order to enhance them. A significant and inverse relationship between self-efficacy and anger control was proved (Lombardo, et al., 2005). Zimmerman correspondingly reached to this conclusion that self-efficacy can have an effect on learning, emotional response, as well as students’ selection of behavior (Zimmerman, 2000). Zareban also in a series of studies on type-II diabetic patients concluded that teaching self-efficacy can lead to a reduction in an individual’s blood sugar level (Zareban, Niknami, & Rakhshani, 2013).
